# Deciphering the Reactivity of Autoantibodies Directed against the RNP-A, -C and 70 kDa Components of the U1-snRNP Complex: “Double or Nothing”?

**DOI:** 10.3390/biomedicines12071552

**Published:** 2024-07-12

**Authors:** Daniel Bertin, Benjamin Babacci, Alexandre Brodovitch, Cléa Dubrou, Xavier Heim, Jean Louis Mege, Nathalie Bardin

**Affiliations:** 1 Service d’Immunologie, Biogénopôle, Hôpital de la Timone, Assistance Publique-Hôpitaux de Marseille (AP-HM), 13005 Marseille, France; 2 Aix Marseille Univ, INSERM, INRAE, C2VN, 13005 Marseille, France; 3 Aix-Marseille Univ, CNRS, ADES UMR 7268, 13005 Marseille, France

**Keywords:** U1-snRNP, RNP (70, A, C), 40 kDa RNP-70 fragment, autoantibodies, mixed connective tissue disease, systemic lupus erythematosus

## Abstract

**Background:** The positivity of anti-RNP autoantibodies as biological criteria for the diagnosis of mixed connective tissue disease (MCTD) has recently divided the rheumatology community. Autoantigenicity of the U1-snRNP complex tends to generate multiple autoantibodies against RNP-A, -C and -70 KDa or Sm proteins. The aim of this study is to identify the most informative autoantibodies in clinical practice, in particular, to contribute to differential diagnosis between MCTD and systemic lupus erythematosus (SLE). **Methods:** Sera from 74 patients positive for anti-RNP autoantibodies were selected over a period of one year of laboratory practice. Autoantibodies directed against extractable nuclear antigen, RNP proteins (A, C, 70 KDa) and 40 kDa fragments of RNP-70 KDa were investigated by using quantitative fluoroenzymatic assay and Western blot analysis. **Results:** Among the 74 patients, 40 patients were diagnosed with SLE, 20 with MCTD, six with another autoimmune disease, three with SARS-CoV-2 infection, three with cancer and two were healthy. No preferential clinical association of IgG or IgM autoantibodies directed against each of the RNP proteins was found between SLE and MCTD. In contrast, the proportion of autoantibodies directed against the RNP component within the U1-snRNP complex showed a significantly higher RNP index in patients with MCTD than in those with SLE (*p* = 0.011), with good performance (sensitivity: 69.2%, specificity: 88.9%). **Conclusions:** The analysis of the proportion of the different autoantibodies directed against the U1-snRNP complex is more informative than the analysis of each autoantibody separately. A follow-up of patients could be informative about the interest of the RNP index as a predictor of disease evolution.

## 1. Introduction

The diagnosis of mixed connective tissue disease (MCTD) remains a major challenge today. Autoantibodies directed against RNP (anti-RNP) and against Sm (anti-Sm), which belong to the family of antinuclear autoantibodies (ANAs), are of paramount importance in clinical practice. Hence, the positivity of anti-RNP is the only biological criterion for mixed connective tissue disease (MCTD) [1], but it is also detected in other autoimmune diseases such as systemic lupus erythematosus (SLE), scleroderma (SSc), Gougerot-Sjögren syndrome (GSS), rheumatoid arthritis (RA) and polymyositis/dermatomyositis (PM/DM) [2]. Anti-Sm are included in the serological criteria of SLE [3] because of their high specificity.

These autoantibodies are directed against small nuclear molecules formed by the association between polypeptides and ribonucleic acid. Anti-RNP interacts with proteins (70 kDa, A, C) that are associated with U1-RNA and Sm proteins to form U1 small nuclear ribonucleoprotein (U1-snRNP) [4]. Anti-Sm antibodies are directed against seven proteins, including B, B’ and D3, which form the common core of small nuclear ribonucleoprotein particles. RNPs are components of the spliceosome, an RNP complex composed of the five snRNPs [5]. The spliceosome is involved in the splicing of precursor mRNA (pre-mRNA) into mature mRNA, ready for translation into proteins. The targets of anti-RNP consist of three proteins, i.e., RNP-70, RNP-A and RNP-C, with some structural and functional differences. Briefly, RNP-70 presents a greater length. Both RNP-A and RNP-70 are capable of direct RNA binding because, unlike RNP-C, they possess an RNA recognition motif [6]. In terms of their functions, RNP-70 initiates spliceosome assembly by early binding to the 5′ splice site of the pre-mRNA and also allows RNP-C to bind to the U1-RNP. RNP-A modulates the initiation of polyadenylation while RNP-C allows for the formation of a transient complex of the spliceosome and stabilizes the RNA/U1-snRNP interaction [7]. During apoptosis, RNP-70 is specifically cleaved by the enzyme caspase-3, resulting in a C-terminally truncated 40 kDa fragment [8,9] which can be targeted by autoantibodies that are more specifically associated with MCTD than other anti-RNP-70 autoantibodies [10].

Several classification criteria have been proposed for the diagnosis of MCTD, all of which include the detection of anti-RNP as the sole serological criterion [1,11,12]. However, the following question remains: what is the added value of subtyping anti-RNP autoantibodies?

According to clinical interest, the detection of anti-RNP is currently carried out in the immunology laboratory and includes an analysis step by indirect immunofluorescence followed, if positive, by the identification of the nuclear target [13,14]. Routinely, anti-RNP autoantibodies are directed against a mixture of RNP-70, RNP-A and RNP-C and belong to the IgG isotype, which is the one usually analyzed [15]. The interest of subtyping anti-RNP autoantibodies for a faster and more specific diagnosis has not yet been established because of conflicting studies in the literature.

The aim of this study was to test and identify the most contributory anti-RNP autoantibodies subtype in clinical practice, especially to aid in the differential diagnosis between MCTD and SLE. The results were then analyzed in relation to the patients’ clinical and immunological data.

## 2. Material and Methods

### 2.1. Patients

All patients with suspected autoimmune diseases who were referred for ANA testing to the Immunology Laboratory of the University Hospital of Marseille between 1 February 2020 and 1 February 2021, and who were positive for IgG anti-RNP autoantibodies, were retrospectively included in this study. Patients with less than 100 µL of serum were excluded.

### 2.2. Clinical Data

This retrospective study exclusively analyzed data issued from health care, and all serum samples were part of a declared Biobank (DC 2012_1704). This study was approved by the Medical Evaluation Board and Health Data Committee of Assistance Publique-Hôpitaux de Marseille, Marseille, France (under GDPR number 2021-108), and fulfilled local requirements for data collection and data protection.

Based on information collected from medical records, 74 patients were classified into five clinical groups according to their disease, including cancer, infectious diseases, SLE, MCTD and other autoimmune diseases. SLE patients met the 2019 EULAR/ACR classification criteria [3], and MCTD patients met the Alarcón–Segovia criteria [1].

### 2.3. Immunoassays

#### 2.3.1. ANA Testing

ANAs in patients’ sera were detected using a commercially available ANA HEp-2 indirect immunofluorescence assay (Kallestad HEp-2 Cell Line Substrate, 12-well slides, Bio-Rad Laboratories, Hercules, CA, USA). ANAs were visually assessed by two experienced observers using a fluorescence microscope (Leica DM-2000, Leica Microsystems, Wetzlar, Germany). The fluorescence pattern and titer were recorded for each sample.

#### 2.3.2. Extractable Nuclear Antigen Antibody Testing

Fluorescence enzyme immunoassays (EliA^™^, Phadia 250, Thermo-Fisher Scientific, Phadia AB, Uppsala, Sweden) were used according to the manufacturer’s instructions to detect IgG autoantibodies directed against RNP, dsDNA, SSA/Ro, SSB/la, SmD, centromere B, Scl-70 and Jo-1 nuclear autoantigens. They were also used to detect IgG and IgM autoantibodies directed against RNP components, namely, RNP-A, RNP-C, RNP-70 and SmBB’ antigens. IgG EliA™ are commercially available and comply with the European In-vitro Diagnostics Regulation (IVDR), whereas IgM EliA™ have been developed by the manufacturer for research purposes only. Fluorescence was quantified using a calibration curve, and the results were expressed in U/mL. For samples with high levels of IgG anti-RNP-A, -RNP-C, -RNP-70, -SmD or -SmBB’ autoantibodies (outside the quantification range), the serum was diluted until the concentration was within the quantification range of the method. A cut-off of 10 U/mL was used for all autoantibodies except for anti-dsDNA IgG and anti-SmBB’ IgG/IgM, where the cut-off was 15 U/mL and 40 U/mL, respectively.

#### 2.3.3. Testing the 40 kDa Fragment of RNP-70

Nuclear extracts from HeLa cells were prepared and separated by SDS-polyacrylamide gel electrophoresis and, subsequently, proteins from the extracts were transferred to a nitrocellulose membrane by Western blotting and cut into strips for the analysis of patient sera. Blocked strips were incubated with 1:100 diluted patient serum in a casein hydrolysate solution. After washing in PBS-Tween, the blots were incubated for one hour with a 1:30,000 diluted alkaline phosphatase-labeled goat anti-human IgG. After washing, the blot strips were developed with NBT/BCIP solution. The molecular weight of the 40 kDa fragment of RNP-70 was determined by alignment and comparison with the bands on the standard blot strips.

### 2.4. Calculation of the RNP Index

To further investigate the proportions of IgG autoantibodies directed against the RNP and Sm components of the U1-snRNP complex, and to compare them between SLE and MCTD, we combined the serum levels of anti-RNP-A, -C, -70 (aRNP-A, -C, -70), anti-SmD (aSmD) and SmBB’ (aSmBB) IgG autoantibodies into a single RNP index (RNPi), calculated as follows:

$$RNPi=\frac{\sum_{j\in \left\{A,C,70\right\}}[aRNP_{j}]}{\sum_{j\in \{A,C,70\}}[aRNP_{j}]+\sum_{k\in \{D,BB^{\prime }\}}\left[aSm_{k}\right]}$$

### 2.5. Statistical Analysis

Statistical analysis was performed using R software version 3.6.0 (R Foundation for Statistical Computing). Continuous variables were compared using Student’s *t*-test when appropriate (otherwise, the Mann–Whitney test), and categorical data were compared using the χ^2^ test when appropriate (otherwise, the Fisher test). Odds ratios (and their 95% confidence intervals) were estimated for each variable using univariate logistic regression. All tests were two-tailed. *p* < 0.05 was retained for significance.

## 3. Results

### 3.1. Clinical Description of Patients Positive for Anti-RNP

**Figure 1:**
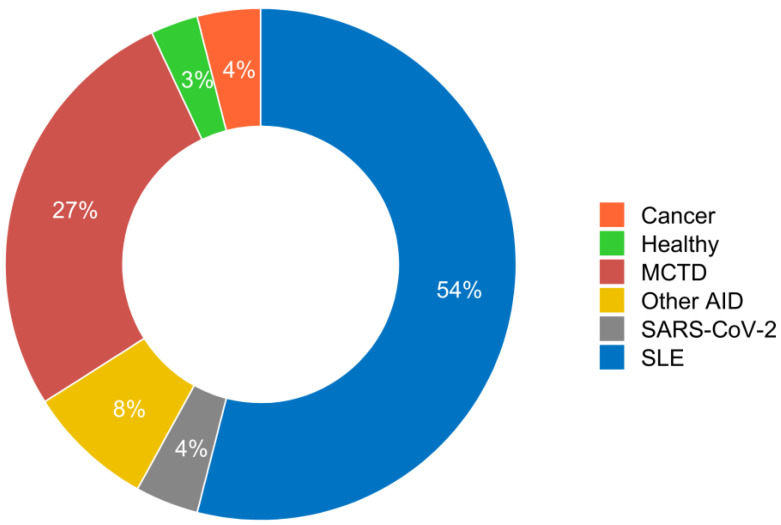
**Distribution of diseases in patients positive for anti-RNP autoantibodies (n = 74).** SLE: systemic lupus erythematosus, MCTD: mixed connective tissue disease, AID: autoimmune disease.

Seventy-four patients who were positive for RNP were included. There were 10 men (13.5%) and 64 women (86.5%) with a mean age of 45.2 ± 16.8 years. Clinically, 40 patients (54%) were diagnosed with SLE and 20 (27%) with MCTD. In addition, six patients (8.1%) had other autoimmune diseases (including one dermatomyositis, one psoriatic arthritis, one systemic sclerosis, one Hashimoto’s disease and two undifferentiated connective tissue disease), three patients (4.05%) had SARS-CoV-2 infection, three patients (4%) had cancer and two patients (2.7%) were classified as healthy with no suspected associated disease (Figure 1). Because of the small number of patients in the other groups, the statistical comparison for the subsequent results will be made only between patients with SLE and those with MCTD. Organ involvement in MCTD and SLE patients is described in Supplementary Table S1.

### 3.2. Differential Diagnosis between MCTD and SLE Based on Immunological Data

The immunological data initially analyzed included anti-nuclear autoantibodies (AAN), IgG anti-dsDNA autoantibodies, IgG autoantibodies against extractable nuclear antigens (ENA), including autoantibodies directed against RNP, SSA/Ro (60 kDa), SSB/La, Sm, centromere, Scl-70 and Jo-1. Both qualitative (Table 1) and quantitative (Table 2) analyses of the results showed a significant difference between patients with MCTD and SLE regarding anti-dsDNA IgG and anti-Sm IgG autoantibody positivity. The prevalence (*p* = 0.011 with OR = 6.49 and *p* = 0.011 OR = 5.67, respectively) and titers (*p* < 0.0001 and *p* = 0.005, respectively) of these autoantibodies were significantly higher in patients with SLE than in patients with MCTD. No difference in anti-RNP levels (whatever their subtype) was observed between patients with SLE and those with MCTD (Table 1). Irrespective of the group of patients, we can note the absence of autoantibodies against centromere, Scl-70 and Jo-1.

### 3.3. Differential Diagnosis between MCTD and SLE Based on the Detection of Autoantibodies against RNP Components

The detection of IgG and IgM autoantibodies directed against RNP components 70, A and C was then performed by the fluoroimmunoenzymatic assay. The detection of IgG autoantibodies against a 40 kDa fragment of RNP-70 was performed by a Western blot assay.

Irrespective of the quantitative (Table 1) or qualitative (Table 2) analyses, no difference was found between patients with SLE and MCTD regarding autoantibodies against RNP proteins (or 40 kDa RNP-70 fragment).

**Figure 2:**
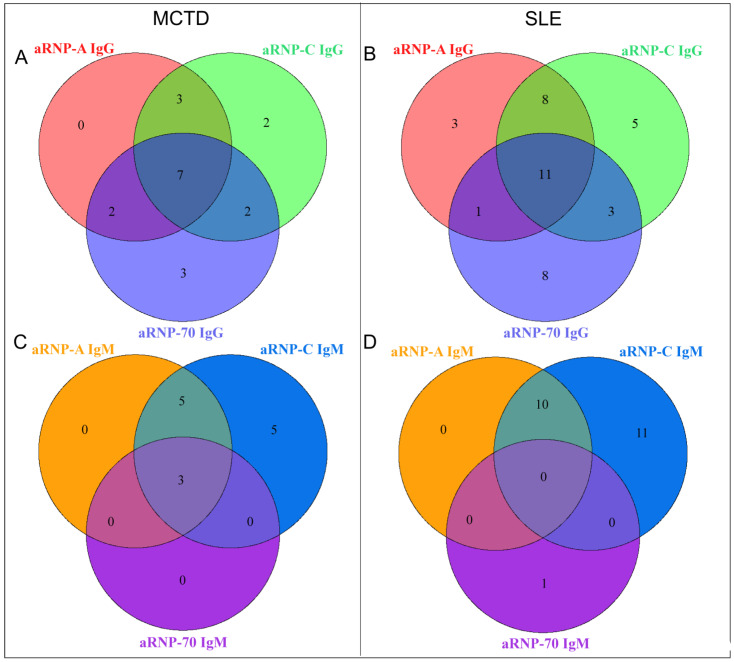
**Serological profiles of anti-RNP autoantibodies for SLE and MCTD patients.** Repartition of MCTD patients according to their positivity of anti-RNP-A/C/70 KDa IgG (**A**) or IgM (**C**). Repartition of SLE patients according to their positivity of anti-RNP-A/C/70 KDa IgG (**B**) or IgM (**D**). aRNP: anti-RNP autoantibody, SLE: systemic lupus erythematosus, MCTD: mixed connective tissue disease.

**Figure 3:**
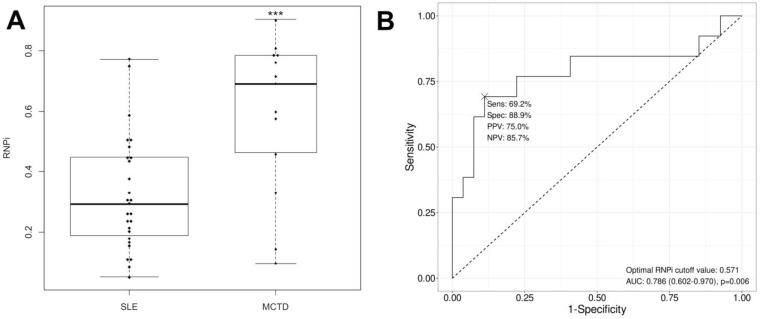
**Performance of RNPi at differentiating MCTD from SLE.** (**A**) Comparison of RNPi between MCTD and SLE patients positive for anti-RNP and anti-Sm autoantibodies. The box plots show the median value and range from the first to the third quartile. The whiskers extend between the maximum and the minimum. RNPi were significantly higher in MCTD patients than in SLE patients (*** *p* < 0.001, Mann–Whitney *U*-test). (**B**) Determination of the cut-off value for RNPi with Receiver Operating Characteristic (ROC) curve (solid line). The dashed line represents the ROC curve for a random guess. Area under curve (AUC) = 0.786; 95% confidence intervals of AUC: 0.602–0.970. Sens: sensitivity, Spec: specificity, PPV: positive predictive value, and NPV: negative predictive value. The best-calculated RNPi cut-off for differentiating MCTD from SLE is 0.571. Patients with RNPi above 0.571 are more likely to have MCTD than SLE.

To further analyze the results, we attempted to combine the results obtained for RNP proteins. We first performed a study of different serological profiles against RNP components, exploring all possible associations, as shown in Figure 2. No difference was found between patients with SLE and MCTD (Figure 2). We then proposed an RNP index calculation, RNPi, as described in Section 2 “Materials and Methods”, which takes into account the RNP and Sm components within the U1-snRNP complex (Table 2). Interestingly, RNPi was significantly higher in MCTD patients than in SLE patients (*p* = 0.011, Table 1), and this result was also found in a subset of patients who were both anti-Sm- and anti-RNP-positive (*p* < 0.001, Figure 3A). In this subset of patients, RNPi above 0.571 discriminated MCTD from SLE with good performance (sensitivity: 69.2%, specificity: 88.9%, positive predictive value: 75%, negative predictive value: 85.7%, Figure 3B).

## 4. Discussion

Autoantigenicity of the U1-snRNP complex generates several autoantibodies against RNP-70, RNP-A, RNP-C and Sm proteins [16]. In order to discriminate between patients with MCTD and SLE, we propose the calculation of an original RNP index (RNPi), reflecting the proportion of autoantibodies produced against the different components within the U1-snRNP complex. This index, which is significantly higher in MCTD than in SLE, indicates a major autoantigenicity against the RNP components in MCTD and constitutes an attractive tool for its diagnosis. This ability of RNPi to discriminate between MCTD and lupus is also found in the subset of patients with both anti-Sm and anti-RNP, in whom it might be expected that the diseases would be more difficult to distinguish due to the presence of biological markers for both diseases.

In a well-documented cohort of patients selected on the basis of anti-U1-snRNP positivity, we found that 27% were diagnosed with MCTD, 54% with SLE and other clinical conditions as reported in the literature [2,17]. The immunological data initially analyzed showed a lack of autoantibodies against centromere B, Scl-70 and Jo-1 in the MCTD patients, but this has also been reported in MCTD cohorts of similar size [18] and does not prevent patients from having clinical signs of scleroderma or myositis, thus allowing a diagnosis of MCTD to be made. We also showed that anti-dsDNA and anti-Sm autoantibodies are significantly associated with SLE. Our data, in agreement with data in the literature [3], are insufficient for differential diagnosis in certain cases, especially in patients with MCTD who are positive for these discriminating autoantibodies.

In order to improve the diagnosis of MCTD, we investigated autoantibodies selectively directed against the individual RNP components, namely, 70, A and C. We found no preferential clinical association for autoantibodies directed against RNP-70, A or C, irrespective of the IgG or IgM isotype, nor for IgG autoantibodies directed against the 40 kDa fragment of RNP-70. The few studies reported in the literature show inconsistent results. Older studies described a preferential association with anti-RNP-70 in MCTD, whereas anti-RNP-A/C was associated with SLE [19,20,21]. Recently, Ahmad et al. concluded that the detection of anti-RNP-70 is not of clinical interest [22]. In addition, previous studies have shown that high levels of anti-RNP are associated with MCTD. In contrast, we have shown that anti-RNP levels do not contribute to the differential diagnosis. Consistent with this, Alves et al. reported that many patients with high anti-RNP levels did not have MCTD, whereas patients with MCTD had either low levels or no evidence of anti-RNP [23]. Additionally, some authors [24,25] reported that IgM anti-U1 snRNP titers were significantly higher in the SLE population than in the MCTD population, but we did not find such a result for the IgM isotype. These inconsistent results may be related to the number of patients and the techniques used. 

**Figure 4:**
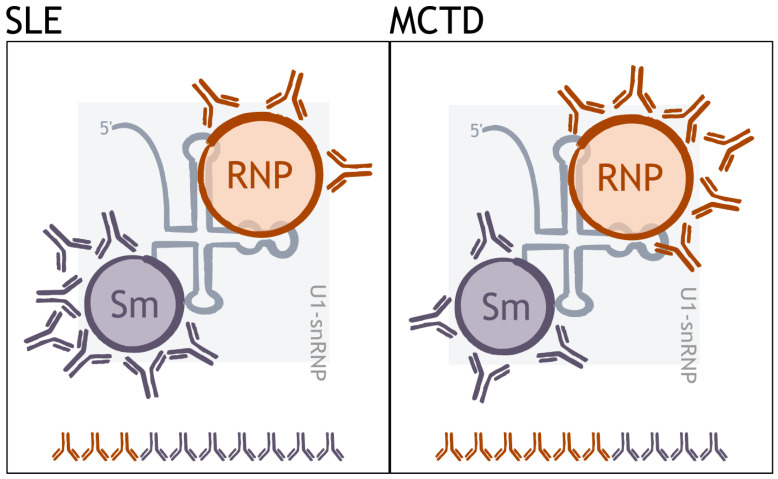
**The proportion of autoantibodies directed against components of the U1-snRNP complex differs between MCTD and SLE**. Among the anti-U1-snRNP autoantibodies, the proportion of autoantibodies directed against the RNP part of the ribonucleoprotein complex is higher in MCTD than SLE. SLE: systemic lupus erythematosus, MCTD: mixed connective tissue disease.

In our study, the evaluation of different serological profiles of the anti-RNP autoantibodies did not reveal any clinical association. In contrast, the proportion of autoantibodies directed against the RNP component within the U1-snRNP complex showed a significantly higher RNP index in patients with MCTD than in those with SLE (Figure 4). This result demonstrates a higher autoimmunogenicity of RNP proteins in MCTD than in SLE. The use of anti-RNP positivity as a criterion for MCTD has recently divided the rheumatology community. Some studies have questioned the relevance of these autoantibodies, as patients without anti-RNP and with typical symptoms have been reported in the literature [23,26]. In addition, a genetic association between the HLA haplotype and anti-RNP autoantibodies has been reported in MCTD, confirming the interest in detecting these autoantibodies [27,28]. In line with this, our results highlight a preferential autoantigenicity against RNP components in MCTD compared to SLE and raise the question of the evaluation of autoantibodies. Which autoantibodies against RNPs should we be looking for? The data suggest that because autoantibodies are produced against a multimolecular complex appearing as a true autoantigenic mosaic, the analysis of each autoantibody separately is not appropriate; rather, the analysis of the proportion of autoantibodies produced against the components of the U1-snRNP complex is more informative.

A major limitation of our study is the size of the cohort. Further studies are needed to test the RNP index in a larger cohort of patients to confirm the diagnosis of MCTD. Patient follow-up could be informative about the interest of this index as a predictor of the evolution of the disease.

## Figures and Tables

**Table 1:** **Comparison** **of quantitative data between the SLE and MCTD groups.**

	Pathology	N	MD	Mean	CI (95%)		Median	STD	IQR	Range		*p*-Value
					Min	Max				Min	Max	
Patient characteristics												
Age at sampling	MCTD	20	0	46.900	37.150	56.650	42.500	20.832	25.750	16	95	0.138 *
	SLE	40	0	40.125	35.698	44.552	41.500	13.844	21.250	14	69	
Age at diagnosis	MCTD	20	0	38.320	28.013	48.627	29.518	22.022	30.691	11.422	87.447	0.354
	SLE	40	0	30.016	26.488	33.544	27.157	11.032	16.199	13.301	59.948	
ANA titer ^†^	MCTD	20	0	4.550	4.108	4.992	5.000	0.945	1.000	1	5	0.850
	SLE	40	0	4.600	4.331	4.869	5.000	0.841	1.000	1	5	
EliA™ IgG												
Anti-U1-snRNP IgG	MCTD	17	3	73.647	48.055	99.239	66.000	49.776	50.000	12.000	179.000	0.553
	SLE	40	0	97.300	72.526	122.074	76.500	77.463	125.000	11.000	273.000	
Anti-U1-RNP-A IgG	MCTD	20	0	94.365	4.647	184.083	15.000	191.698	66.050	1.000	750.000	0.869
	SLE	40	0	38.898	21.794	56.001	19.500	53.479	46.650	0.600	227.000	
Anti-U1-RNP-C IgG	MCTD	20	0	76.435	0.000	161.866	19.000	182.540	58.275	1.400	834.000	0.666
	SLE	40	0	80.248	44.870	115.625	37.000	110.619	86.050	1.300	394.000	
Anti-U1-RNP-70 IgG	MCTD	20	0	152.420	0.000	329.234	61.000	377.796	125.900	0.300	1730.00	0.456
	SLE	40	0	61.722	37.526	85.919	21.000	75.658	108.525	0.300	265.000	
Anti-SmDP-S, IgG	MCTD	20	0	33.935	0.000	88.945	0.900	117.539	1.975	0.700	520.000	0.005
	SLE	40	0	104.483	44.785	164.180	9.650	186.662	103.100	0.700	790.000	
Anti-Ro, IgG	MCTD	20	0	16.345	0.000	35.604	0.900	41.151	5.750	0.300	149.000	0.843
	SLE	34	6	13.253	2.831	23.674	0.900	29.868	1.875	0.300	115.000	
Anti-La, IgG	MCTD	20	0	0.625	0.363	0.887	0.500	0.560	0.325	0.300	2.800	0.712
	SLE	38	2	1.045	0.419	1.671	0.450	1.905	0.400	0.300	9.500	
Anti-Scl-70s, IgG	MCTD	20	0	0.830	0.654	1.006	0.650	0.376	0.225	0.600	1.900	0.048
	SLE	40	0	1.315	0.830	1.800	0.900	1.517	0.725	0.600	9.700	
Anti-CENPB, IgG	MCTD	20	0	0.555	0.435	0.675	0.400	0.256	0.225	0.400	1.300	0.340
	SLE	40	0	0.690	0.547	0.833	0.500	0.446	0.425	0.300	2.500	
Anti-Jo-1, IgG	MCTD	20	0	0.395	0.306	0.484	0.300	0.190	0.125	0.300	1.100	0.819
	SLE	40	0	0.402	0.333	0.473	0.300	0.219	0.100	0.300	1.400	
Anti-SmBB’, IgG	MCTD	20	0	136.745	24.290	249.200	45.500	240.280	52.250	5.900	980.000	0.480
	SLE	40	0	394.882	178.809	610.956	81.500	675.619	458.750	1.600	3400.000	
Anti-dsDNA IgG	MCTD	14	6	6.379	1.608	11.149	1.300	8.262	7.425	0.500	24.000	<0.001
	SLE	33	7	36.864	24.699	49.028	26.000	34.307	55.200	0.600	110.000	
EliA™ IgM												
Anti-U1-RNP-A, IgM	MCTD	20	0	51.495	0.000	110.114	4.200	125.251	18.775	0.100	462.000	0.147
	SLE	40	0	13.473	1.642	25.303	2.050	36.991	7.775	0.000	224.000	
Anti-U1-RNP-C, IgM	MCTD	20	0	69.995	0.000	168.618	20.500	210.727	23.950	0.200	959.000	0.546
	SLE	40	0	23.892	11.618	36.167	12.500	38.381	21.300	0.600	211.000	
Anti-U1-RNP-70, IgM	MCTD	20	0	11.465	0.000	25.919	0.600	30.884	1.675	0.000	131.000	0.063
	SLE	40	0	1.093	0.348	1.837	0.300	2.328	1.150	0.000	13.0000	
Anti-SmBB’, IgM	MCTD	19	1	49.211	35.508	62.914	39.000	28.430	33.500	18.000	108.000	0.981
	SLE	40	0	97.325	38.641	156.009	33.500	183.494	53.250	12.000	912.000	
Calculated RNP Index												
RNPi	MCTD	20	0	0.673	0.559	0.787	0.759	0.243	0.216	0.095	0.972	0.011 *
	SLE	40	0	0.479	0.389	0.569	0.449	0.282	0.541	0.053	0.945	

SLE: systemic lupus erythematosus, MCTD: mixed connective tissue disease, CI: confidence interval, STD: standard deviation, IQR: inter-quartile range, MD: missing data. Mann–Whitney U-test except for *p*-values marked with an asterisk (*) where Student *t*-test was performed. (^†^) ANA titers were arbitrarily coded by integers from 1 to 5 corresponding to titers from 1:160 to >1:1280, respectively.

**Table 2:** **Comparison** **of qualitative data between the SLE and MCTD groups.**

	MCTD n = 20	SLE n = 40	*p*-Values	Odd Ratio [95% CI]
**Demographical data:**				
**Gender:**				
Men	1 (5%)	5 (12.5%)	0.653	0.368 [0.040–3.390]
Women	19 (95%)	35 (87.5%)		
**Serological data: IIF (HEp-2)**				
**ANA titer:**				
Titer ≤ 320	1 (5%)	2 (5%)	1.000	1.000 [0.0852–11.7]
Titer > 320	19 (95%)	38 (95%)		
**ANA pattern:**				
Speckled	2 (10%)	12 (31.6%)	0.106	0.241 [0.0480–1.21]
Speckled-Homogenous	18 (90%)	26 (68.4%)		
**Serological data: EliA™ IgG**				
**Anti-U1-RNP-A IgG**				
Negative	8 (40%)	17 (42.5%)	0.853	0.902 [0.303–2.69]
Positive	12 (60%)	23 (57.5%)		
**Anti-U1-RNP-C IgG**				
Negative	6 (30%)	13 (32.5%)	0.844	0.890 [0.278–2.85]
Positive	14 (70%)	27 (67.5%)		
**Anti-U1-RNP-70 IgG**				
Negative	6 (30%)	17 (42.5%)	0.348	0.580 [0.185–1.82]
Positive	14 (70%)	23 (57.5%)		
**Anti-SmDP-S, IgG**				
Negative	17 (85%)	20 (50%)	**0.011**	**5.67 [1.43**–**22.4]**
Positive	3 (15%)	20 (50%)		
**Anti-SmBB’ IgG**				
Negative	7 (35%)	16 (40%)	0.707	0.808 [0.265–2.46]
Positive	13 (65%)	24 (60%)		
**Anti-Ro, IgG**				
Negative	16 (80%)	28 (70%)	0.541	1.71 [0.473–6.21]
Positive	4 (20%)	12 (30%)		
**Anti-La, IgG**				
Negative	20 (100%)	38 (95%)	0.548	2.66 [0.122–58.1]
Positive	0 (0%)	2 (5%)		
**Other Anti-ENA IgG**				
Negative	6 (30%)	11(27.5%)	0.839	1.13 [0.347–3.68]
Positive	14 (70%)	29 (72.5%)		
**Anti-dsDNA IgG**				
Negative	11 (78.6%)	13 (36.1%)	**0.011**	**6.49 [1.53**–**27.6]**
Positive	3 (21.4%)	23 (63.9%)		
**Serological data: EliA™ IgM**				
**Anti-U1-RNP-A, IgM**				
Negative	12 (60%)	30 (75%)	0.232	0.500 [0.159–1.57]
Positive	8 (40%)	10 (25%)		
**Anti-U1-RNP-C, IgM**				
Negative	7 (35%)	19 (47.5%)	0.357	0.595 [0.196-1.80]
Positive	13 (65%)	21 (52.5%)		
**Anti-U1-RNP-70, IgM**				
Negative	17 (85%)	39 (97.5%)	0.103	0.145 [0.0141–1.50]
Positive	3 (15%)	1 (2.5%)		
**Anti-SmBB’, IgM**				
Negative	10 (50%)	22 (55%)	0.714	0.818 [0.279–2.40]
Positive	10 (50%)	18 (45%)		
**Serological data: Western-blot**				
**Anti-RNP 40 kDa, IgG**				
Negative	14 (70%)	27 (67.5%)	0.844	1.12 [0.351–3.59]
Positive	6 (30%)	13 (32.5%)		

IIF: indirect immunofluorescence, ANA: anti-nuclear autoantibodies, ENA: extractable nuclear antigen, CI: confidence interval. “Other Anti-ENA IgG” category compute qualitative result of all tested IgG autoantibodies with the exception of anti-RNP A/C/70 KDa/40 KDa IgG. The chi-square test was used to compare categorical variables (Fisher’s exact test was used if chi-square test was not applicable).

## Data Availability

The data will be shared on reasonable request to the corresponding author after obtaining permission from Assitance Publique Hopitaux de Marseille.

## Supplementary Materials

The following supporting information can be downloaded at: https://www.mdpi.com/article/10.3390/biomedicines12071552/s1, Table S1: Organ involvement in SLE and MCTD groups.
