# Supplementary material for: Deciphering the Reactivity of Autoantibodies Directed against the RNP-A, -C and 70 kDa Components of the U1-snRNP Complex: “Double or Nothing”?
Source: Biomedicines. 2024 Jul 12;12(7):1552. doi: 10.3390/biomedicines12071552 (PMC11275026; doi:10.3390/biomedicines12071552)
Supplement: Supplementary file 1 [file biomedicines-12-01552-s001.zip › biomedicines-3048957-supplementary.pdf]

Table S1: Organ involvement in SLE and MCTD groups

**Organ involvement**	**SLE (n=40)**	**MCTD (n=20)**
Articular	33 (82.5%)	18 (90%)
Cutaneous	19 (47.5%)	11 (55%)
Renal	23 (57.5%)	3 (15%)
Microvascular (Raynaud phenomenon)	14 (35%)	12 (60%)
Muscular	2 (5%)	6 (30%)
Cardiac	10 (25%)	3 (15%)
Pulmonar	3 (7.5%)	6 (30%)
Neurological	1 (2.5%)	1 (5%)
Hematologic	5 (12%)	0 (0%)
Hepatic	1 (2.5%)	2 (10%)
Digestive (Eosophagus)	0 (0%)	3 (15%)
